# Combination of Quercetin and Vitamin E Supplementation Promotes Yolk Precursor Synthesis and Follicle Development in Aging Breeder Hens via Liver–Blood–Ovary Signal Axis

**DOI:** 10.3390/ani11071915

**Published:** 2021-06-28

**Authors:** Felix Kwame Amevor, Zhifu Cui, Xiaxia Du, Zifan Ning, Gang Shu, Ningning Jin, Xun Deng, Yaofu Tian, Zhichao Zhang, Xincheng Kang, Dan Xu, Guishuang You, Yao Zhang, Diyan Li, Yan Wang, Qing Zhu, Xiaoling Zhao

**Affiliations:** 1 Farm Animal Genetic Resources Exploration and Innovation Key Laboratory of Sichuan Province, Sichuan Agricultural University, Chengdu 611130, China; amevorfelix@gmail.com (F.K.A.); 2018102013@stu.sicau.edu.cn (Z.C.); 15680811065@163.com (X.D.); ningzifan@163.com (Z.N.); jnn233333@163.com (N.J.); dengxun082439@163.com (X.D.); maikurakitt@163.com (Y.T.); Tiger@sicau.edu.cn (Z.Z.); kangxincheng@stu.sicau.edu.cn (X.K.); youguishuang@stu.sicau.edu.cn (G.Y.); zhangyao12316@163.com (Y.Z.); diyanli@sicau.edu.cn (D.L.); as519723614@163.com (Y.W.); zhuqingsicau@163.com (Q.Z.); 2 Department of Basic Veterinary Medicine, Sichuan Agricultural University, Chengdu 611130, China; dyysg2005@sicau.edu.cn (G.S.); 13086605395@163.com (D.X.)

**Keywords:** quercetin, vitamin E, yolk precursor synthesis, liver–blood–ovary signal axis, aging breeder chicken

## Abstract

**Simple Summary:**

This study evaluated the capacity of dietary quercetin, vitamin E and their combination to promote follicle development and attenuate organ inflammation by improving the antioxidant capacity of the liver–blood–ovary signal axis of aging broiler breeder hens. The results from this study showed that the combination of quercetin and vitamin E synergistically improved the chicken’s reproductive organ characteristics, and also showed protective effects on liver morphology and histology. Moreover, the antioxidant parameters, reproductive hormones and receptors, liver lipid synthesis, and the levels of mRNAs related to yolk precursor synthesis (very low density apolipoprotein-II and vitellogenin-II), lipid transport (microsomal triglyceride transport protein), lipogenesis (fatty acid synthase), and follicle developments were increased remarkably by the combination of quercetin and vitamin E. The results obtained in this study provide an important reference for the combination of quercetin and vitamin E as a functional feed additive for promoting the functions of the liver–blood–ovary axis, and also as a potential chemopreventive and chemotherapeutic agent for improving liver and ovary functions in chickens by acting as a hepatoprotective and oviprotective agent. This could facilitate the transport and exchange of synthetic substances (including hormones, yolk precursors, and other biochemical substances) among the liver–blood–ovary alliances to ensure the synchronous development and functional coordination between the liver and ovary in aging breeder hens.

**Abstract:**

The fertility of female animals is negatively correlated with increasing chronological age. In aging broiler breeder hens, there is a decline in the functionality of the ovary and liver accompanied by hormonal or endocrine changes, a reduction in antioxidant capacity, and a decrease in folliculogenesis. Therefore, improving the reproductive function in aging breeder hens using dietary strategies is of great concern to the poultry breeder. This study evaluated the capacity of dietary quercetin (Q), vitamin E (VE), and their combination (Q + VE) to promote follicle development and attenuate organ inflammation by improving the antioxidant capacity of aging breeder hens. In this study, 400 broiler breeder hens (Tianfu broilers breeder hens, 435 days old) were allotted into four groups (100 birds each) with four replicates each (25 birds each). They were fed diets containing Q (0.4 g/kg), VE (0.2 g/kg), Q + VE (0.4 g/kg + 0.2 g/kg), and a basal diet for 10 weeks. The results showed that Q + VE improved the organ characteristics (*p* < 0.05), and also that Q + VE showed protective effects on the liver against injury, as well as increasing the antioxidant capacity of the liver, serum, and ovary (*p* < 0.05). Furthermore, liver lipid synthesis was increased remarkably, as indicated by the changes in triglyceride levels in hens fed Q + VE (*p* < 0.05). Levels of E2, FSH, and LH, their receptors, and mRNAs related to yolk precursor synthesis were increased by the Q + VE (*p* < 0.05). Therefore, the combination of quercetin and vitamin E synergistically promotes and regulates the transportation and exchange of synthetic substances among the liver–blood–ovary alliances to ensure the synchronous development and functional coordination between the liver and ovary in aging breeder hens.

## 1. Introduction 

The fecundity of female animals is negatively correlated with increasing chronological age [1,2,3,4,5]. Broiler breeder hens usually fall into disuse at around 65–70 weeks. After peak egg production at around 30–35 weeks, egg production gradually declines and falls to less than 50% after 60 weeks [3]. Aging in broiler breeder hens is predominantly characterized by a decline in the functionality of the ovary and liver accompanied by hormonal or endocrine changes, a reduction in antioxidant capacity, a decrease in vitellogenesis, lipogenesis, and the transport and accumulation of yolk precursors in the oocyte, a reduction in ovarian follicular reserve with decreased oocyte quality [4,5,6,7,8], a decrease in the volume of hepatocytes and changes in hepatocyte nuclear morphology [9], and the reduction in follicles selected into the preovulatory hierarchy [3,4,5,10]. The mechanisms and correlations within the liver–blood–ovary signal axis largely influence the formation and deposition of yolk precursors into the oocyte of hens, which are mostly regulated by reproductive hormones, including estrogens, that promote follicle development and hence egg production [11,12]. Follicular development is closely associated with changes in the reproductive organs, and the mRNA expression of hormone receptors such as follicle stimulating hormone receptor (FSHR) and luteinizing hormone receptor (LHR) [13]. The liver is responsible for synthesizing yolk precursors (vitellogenin (VTG), apolipoproteins (Apo), triglyceride (TG), phospholipids, and cholesterol) through a process called vitellogenesis and lipogenesis, respectively, primarily under the influence of estrogen, which is mostly produced by the ovary [14]. During peak egg production, the intense vitellogenesis and lipogenesis in the chicken liver result in an increased liver size and change in liver coloration [12,15]. Oxidative stress induces apoptosis in granulosa cells and causes atresia in rats [16], which results in granulosa cell dysfunction and eventually female infertility [17]. Usually, in chickens, oxidative stress reaches its peak when the hen is about 65 to 70 weeks old [3]. Therefore, boosting the antioxidant defense system of these organs, and promoting the endocrine and transcription factors of the aging breeder hens, in order to promote yolk precursor synthesis and follicle formation is a major concern for chicken breeders. Recently, many researchers employed numerous natural plant extracts including lycopene [18], grape seed proanthocyanidin extracts [19], resveratrol [20], and hesperidin [21] to reduce oxidative stress in the reproductive organs, including ovaries, in order to maintain normal function. Dietary daidzein (isoflavone phytoestrogen) was reported to promote laying performance and follicle development in aging laying hens [22,23].

Quercetin (3,3′,4′,5,7-pentahydroxyflavonone) is a ubiquitous dietary flavonoid, which is found in a variety of beverages, fruits and vegetables. It is known to have antioxidant, anti-aging [24,25,26], and phytoestrogenic properties, and has been studied extensively for possible beneficial biological activities, including estrogen-like and estrogen-independent effects, and reactive oxygen species (ROS) scavenging potential [25,27]. Quercetin could regulate enzyme-mediated antioxidant and non-enzyme-dependent antioxidant defense systems, and was also reported to regulate signaling pathways such as Cytochrome C-type protein (NRFB), 5′adenosine monophosphate-activated protein kinase (AMPK), and mitogen-activated protein kinases (MAPK) caused by ROS to promote the antioxidant defense system and maintain oxidative balance in animals [28]. Quercetin as a phytoestrogenic compound (which structurally mimics endogenous estradiol) could compete with estradiol for binding to the estrogen receptors to increase the expression of estrogen-responsive genes [27]. These indicated that quercetin, as a dietary supplement, may promote laying performance and follicle development by increasing the mRNA expression of hormone receptors and transcriptional genes related to follicle development in laying hens [27].

Vitamin E is one of the active natural antioxidants primarily used in animal feeding against cellular free radical damage [29,30]. Reports indicated that dietary vitamin E could improve the reproductive and antioxidant capability of breeder chickens [31,32], as well as protecting the liver against lipid peroxidation, apoptosis, and cell membrane damage [33]. Vitamin E supplementation promotes chicken embryo development by increasing the nutrient composition (tocopherol) and antioxidant stability of chicken eggs [30,34]. Moreover, supplementing vitamin E in laying hens promotes egg production and egg quality by facilitating the release of yolk precursor (vitellogenin) from the liver, as well as acting as an anti-stressor [35,36,37]. This shows that vitamin E could promote yolk precursor synthesis in breeder hens. 

Accumulating evidence has indicated that quercetin and vitamin E promote reproductive and antioxidant functions in breeder hens [27,30]; however, the mechanisms through which the supplements, individually or in combination, actively enhance the reproductive and anti-oxidative functions of aging breeder hens have not yet been established. Therefore, this study investigated the effect of dietary quercetin (Q), vitamin E (VE), and their combination on mechanisms of oxidative stress scavenging, and the promotion of transcriptions and biochemical parameters related to yolk precursor synthesis and hormonal regulation, and follicle development of the aging breeder hens. The results from this study can delineate the underlying mechanisms of the beneficial effects of supplemental quercetin and vitamin E or their combination on the yolk precursor synthesis, follicle development, and antioxidant capacity of aging breeder hens.

## 2. Materials and Methods

### 2.1. Birds, Management, and Experimental Treatment

This study was approved by the Animal Care and Use Committee of Sichuan Agricultural University. Animals used in this experiment were cared for under the guidelines stated in the Guide for the Care and Use of Agricultural Animals in Agricultural Research and Teaching of Sichuan Province, China (No. DKY-2019502005).

A total of 400 Tianfu broiler breeder hens (435 days old, with laying rate >50%) obtained from the Chicken Breeding Unit of Sichuan Agricultural University, at the same facility where the experiment was conducted, and were randomly allotted to 4 experimental groups (100 birds each) with 4 replicates per group and 25 hens per replicate. “Tianfu broiler chicken” is a fast and high-quality jute feathered green foot type quality chicken developed by the Poultry Research Breeding Group of Sichuan Agricultural University and Sichuan Banghe Agricultural Science and Technology Co., Ltd. of China, Sichuan, China. Tianfu broiler breeder hens are a highly productive local indigenous chicken breed, and there are both meat-type and egg-type chickens with fast growth rates. The egg-type breed usually reaches peak egg production (>95%) at around 30–35 weeks of age; however, this declines gradually and falls by less than 60% after 60 weeks [38]. The experimental hens were kept in individual wire cages with the following dimensions: width 48.8 cm, depth 38.1 cm; height 38.1 cm. They were kept under the photoperiod 16 h Light: 8 h Dark, at a temperature of 22 ± 1 °C, with optimal ventilation during the 10-week experimental period. On a daily basis, the hens were inspected for any health-related problems. In this study, the chickens were fed diets containing quercetin at 0.4 g/kg, vitamin E (0.2 g/kg), quercetin and vitamin E (0.4 g/kg and 0.2 g/kg), and a basal diet (control) for a period of 10 weeks. 

The composition and nutrient level of the basal diet are presented in Table 1. 

### 2.2. Clinical Blood Parameters 

At the end of the experiment (10 weeks), after feed deprivation for 12 h, two (2) hens per replicate were randomly selected (*n* = 8 per group) and blood samples were collected from the wing vein (5 mL) of each group. The blood samples were centrifuged at 3000 rpm for 10 min at 4 °C to obtain the serum and then stored at −80 °C until hormone and biochemical analyses were conducted. The levels of estrogen (E2), follicle-stimulating hormone (FSH), and luteinizing hormone (LH) were measured using commercially specific enzyme-linked immunosorbent assay (ELISA) kits following the protocols provided by the manufacturer (Baolai Biotechnology Co., Ltd., Yancheng, China).

### 2.3. Reproductive Organ Characteristics of Aging Breeder Hens

To obtain reproductive organs/organs, the hens (*n* = 8 per group) were weighed and euthanized. We collected and weighed oviduct, ovary, follicle (F1 to F3), abdominal fat, and liver, measured and recorded follicle number (F1-F3; >8 mm in diameter), follicle diameter (mm) (F1 to F3) and then calculated oviduct index, ovary index, liver index and follicular index. The chicken organs were weighed individually and expressed as a percentage of slaughtered weight. The organ index was calculated as organ index = organ weight/hen weight × 100. Parts of the livers and ovaries (ovaries without prehierarchical and preovulatory follicles) samples were collected and immediately snap-frozen in liquid nitrogen and later stored at −80 °C for subsequent RNA extraction and qRT-PCR analysis, and others (liver and ovary samples) were stored at −40 °C for the evaluation of biochemical and antioxidant parameters. Other parts of the liver samples were stored in 4% paraformaldehyde solution for subsequent histological and morphological analyses. Therefore, the liver samples collected were divided into three parts for further analysis (biochemical, RNA extraction, and morphological and histological analyses), whereas the ovary samples collected were divided into two parts for biochemistry and RNA analyses. 

### 2.4. Biochemical Analysis

To measure biochemical parameters, 0.3 g liver and ovarian (ovaries without prehierarchical and preovulatory follicles) tissues were homogenized in 2.7 mL phosphate buffer saline (PBS). The protein concentration in the tissue homogenates was determined using a Total Protein Assay kit (Nanjing Jiancheng Bioengineering Institute, Nanjing, China). The parameters listed below were measured in the serum, liver, and ovary. 

#### 2.4.1. Triglyceride (TG), Total Cholesterol (T-CHO), Aspartate Aminotransferase (AST) and Alanine Aminotransferase (ALT)

The levels of TG and T-CHO were determined in the liver, serum, and ovary using biochemistry commercial kits following the guidelines of the manufacturer (Nanjing Jiancheng Bioengineering Institute, Nanjing, China). Serum AST and ALT levels were determined calorimetrically using standard kits supplied by Baolai Biotechnology Co., Ltd., Yancheng, China. 

#### 2.4.2. Vitellogenin (VTG), and Very Low Density Lipoprotein y (VLDLy)

The concentrations of VTG and VLDLy levels were also measured from the liver, serum, and ovary using enzyme-linked immunosorbent assay (ELISA) kits with guidance from the manufacturer (Baolai Biotechnology Co., Ltd., Yancheng, China).

#### 2.4.3. Oxidative Stress Parameters Assay in the Liver, Serum, and Ovary

We further evaluated the activities of the various antioxidant parameters including: total antioxidant capacity (T-AOC), catalase (CAT), total superoxide dismutase (T-SOD), concentrations of glutathione (GSH), methane dicarboxylic aldehyde (MDA), and glutathione peroxidase (GSH-Px) using commercial kits following the guidelines of the manufacturer (Nanjing Jiancheng Bioengineering Institute, Nanjing, China).

### 2.5. Morphological and Histological Analyses/Histological Staining

The hematoxylin and eosin (HE) staining was performed on the liver tissue using standard protocols previously described by Cui et al. (2020). Thus, the tissues (um/g) were fixed for 24 h and embedded with paraffin. Thereafter, the tissue sections were selected and used for histological and morphological observations. Frozen sections of the liver tissue were carbowax-embedded and oil-red staining was performed, and then all the sections were viewed under fluorescence microscope and photos were taken (DP80Digital, Olympus, Tokyo, Japan). 

### 2.6. RNA Isolation and Quantitative Real-Time PCR (qRT-PCR)

We isolated total RNA from the liver and ovary tissues following the procedures described in our previous study [12], using Trizol reagent (Takara, Dalian, China), and abiding by the instructions provided by the manufacturer. Thereafter, the concentration and purity of the isolated RNA were determined by using Nanodrop 2000C (Thermo Fisher Scientific, Waltham, MA, USA) with the A260/280 absorbance ratio. Furthermore, PrimeScript RT Reagent Kit (Takara, Dalian, China) was used to synthesize the single-strand cDNA following the manufacturer’s instructions. Then, the single-strand cDNA was used for qRT-PCR analysis through the CFX96 Real-Time System (Bio-Rad, Hercules, CA, USA) with the following suitable conditions: 95 °C for 3 min, 40 cycles of 95 °C for 10 s and annealing temperature (Table 2) for 20 s, followed by a final extension at 72 °C for 20 s, with a melt curve analysis performed at 65~95 °C. The amplification efficiencies of target genes ranged from 95% to 105%. Each qRT-PCR reaction was performed with volumes of 15 µL containing 6.25 µL TB Green TM Premix (Takara), 0.3 µL forward and reverse primers, 1.5 µL cDNA, and 6.65 µL DNase/RNase-Free Deionized Water (Tiangen, Beijing, China). All samples were measured in triplicate and the experiment was performed twice. The relative abundance of each transcript was normalized with GAPDH. Gene expression was calculated using the 2^−∆∆Ct^ method [39].

### 2.7. Statistical Analysis

All experimental data were presented as mean ± standard deviation (SD). Analyses were performed with a one-way analysis of variance (ANOVA) followed by the Tukey test using GraphPad Prism 5 (GraphPad Software Inc., San Diego, CA, USA). Values differed significantly at *p* < 0.05. 

To fit a normal distribution, percentages were transformed to arc sines of the square roots. Other data were transformed to common logarithms. Transformations were made before ANOVA. Thus, the experimental data were first tested by normal distribution, and on this basis, we carried out one-way analysis of variance (ANOVA), in which the statistics included the homogeneity test of variance.

## 3. Results

### 3.1. Effects of Quercetin (Q), Vitamin E (VE), and Their Combination (Q + VE) on Organ Characteristics of Aging Breeder Hens

The results presented in Table 3 showed that there was no difference in the live weight and liver index between the control group and all the treatment groups (Q, VE, and Q + VE) (*p* > 0.05). For oviduct index, we observed a significant increase in the Q + VE group as compared to the control group (*p* < 0.05). The length of the oviduct was higher in the quercetin and combination (Q + VE) groups (*p* < 0.05), but the vitamin E group had no difference with the control group (*p* > 0.05). The ovary indexes of all the treatment groups were significantly higher than the control group (*p* < 0.05). No difference existed between all the treatment groups (*p* > 0.05). 

We also observed an increased follicular number for the treatment groups compared to the control group (*p* < 0.05), but no difference existed between the treatment groups (*p* > 0.05). For individual follicular indexes (F1, F2, and F3), we observed that the F1 weight indexes of (V and Q + V) groups were significantly higher compared to the control (*p* < 0.05). For the F1 and F2 follicle indexes, we observed significantly higher values for all the treatment groups as compared to the control group (*p* < 0.05), whereas no difference existed between all the treatment groups (*p* > 0.05). In addition, the follicle diameters of the treatment groups were significantly higher than those of the control (*p* < 0.05); however, the values for the combination (Q + VE) group were significantly higher than those of both the quercetin and vitamin E groups (*p* < 0.05), but no difference existed between the quercetin and vitamin E groups (*p* > 0.05). Moreover, the abdominal fat index was dramatically reduced in the Q + VE group compared to the control, but was not statistically different with the control, Q and VE groups (*p* > 0.05, Table 3). 

Oviduct index = Oviduct weight/live body weight × 100 

Liver index = Liver weight/live body weight × 100

Ovary index = oviduct weight/live body weight × 100 

Follicle F1 index = Follicle F1 weight/live body weight × 100

Follicle F2 index = Follicle F2 weight/live body weight × 100

Follicle F3 index = Follicle F3 weight/live body weight × 100

Abdominal fat index = Abdominal fat weight/live body weight × 100

### 3.2. Effects of Quercetin, Vitamin E, and Their Combination (Q + VE) on Liver Morphological and Histological Changes of Aging Breeder Hens

**Figure 1:**
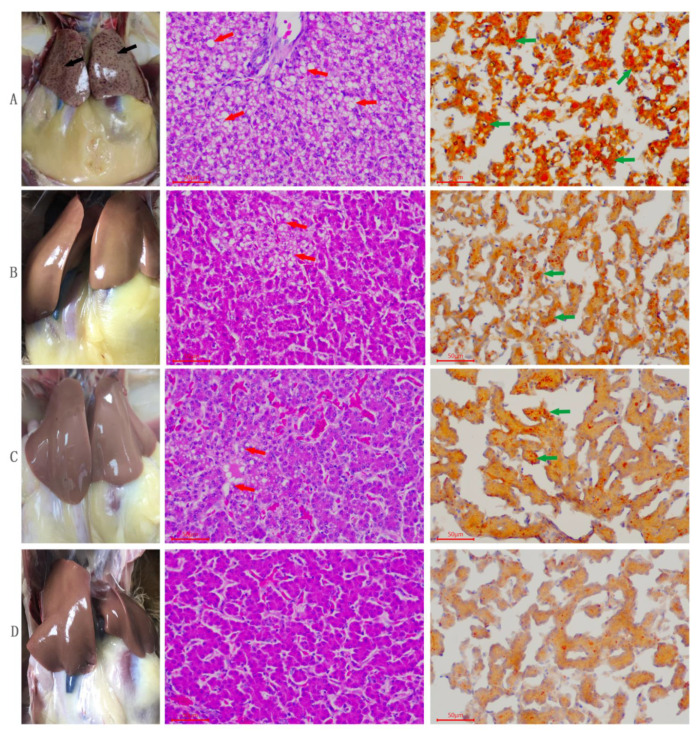
Effects of quercetin, vitamin E, and their combination (Q + VE) on liver morphological and histological changes of aging breeder hens. This figure shows the Hematoxylin-eosin (HE) staining and oil red results of the hen’s liver tissue. (**A**) (Control Group): severe hepatic steatosis. (**B**) (Quercetin Group): mild hepatic steatosis. (**C**) (Vitamin E Group): moderate hepatic steatosis. (**D**) (Q + VE Group): normal liver morphology without any steatosis. The red arrow indicates the “fatty vacuoles”, the green arrow indicates the “lipid droplets”.

The results of histopathology and oil red staining are presented in Figure 1. For the control group, we observed severe pathological changes, characterized by severe steatosis of hepatocytes, which indicated fatty liver condition (Figure 1A). Moreover, the quercetin group showed mild pathological changes in the structure of the liver tissues and mild steatosis in hepatocytes (Figure 1B). The Vitamin E group also showed moderate pathological changes with moderate steatosis in hepatocytes (Figure 1C). Lastly, in the combination group (Q + VE), the liver tissue structure was normal without steatosis (Figure 1D).

### 3.3. Effects of Q, VE, and Q + VE on the Biochemical Changes in the Liver, Serum, and Ovary of the Aging Breeder Hens

#### 3.3.1. TG and TC Levels in the Liver, Serum, and Ovary 

**Figure 2:**
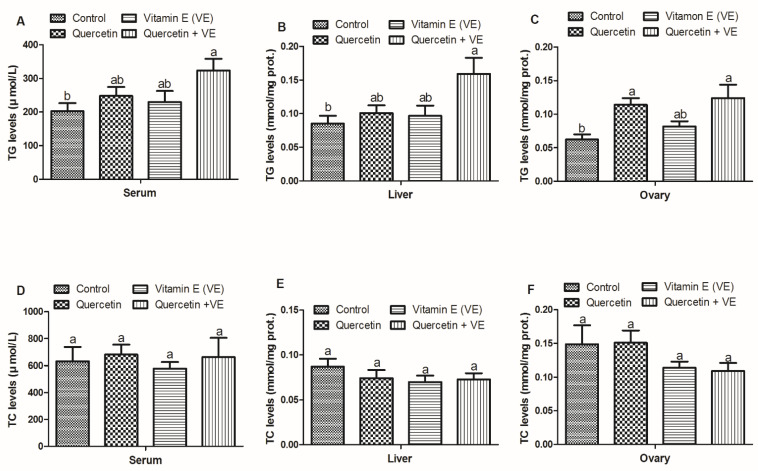
Effects of quercetin, vitamin E, and quercetin + vitamin E on the triglyceride (TG) and total cholesterol (TC) levels in the liver, serum, and ovary of the aging breeder hens. Figure 2: Levels of TG (**A**–**C**) and TC (**D**–**F**). The values are presented as the mean ± SD. Bars without the same letter differed significantly (*p* < 0.05). prot: protein.

As shown in Figure 2, the results for lipid profiles in the serum, liver and ovary were measured with commercial kits. The results showed that TG in the serum and liver of the treatment groups were elevated as compared with the control group; however, the difference existed only between the control and the combination (Q + VE) groups (*p* < 0.05). For the ovary, the level of TG in the vitamin E group was statistically similar to all of the other three treatments (*p* < 0.05). No difference existed between the control and vitamin E groups (*p* > 0.05, Figure 2A–C). 

The TC levels in the liver, serum, and ovary were not significant in all the dietary groups; however, only the quercetin + Vitamin E group is numerically lower than the other treatments (*p* > 0.05, Figure 2D–F). 

#### 3.3.2. VLDLy and VTG Levels in the Liver, Serum, and Ovary 

**Figure 3:**
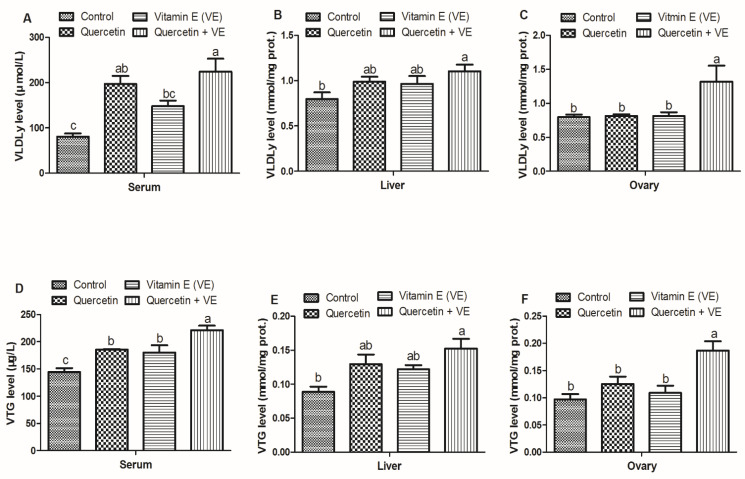
Effects of quercetin, vitamin E, and quercetin + vitamin E on the very low density lipoprotein (VLDLy) and vitellogenin (VTG) levels in the liver, serum, and ovary of the aging breeder hens. prot: protein. Levels of VLDLy (**A**–**C**) and VTG (**D**–**F**). The values are presented as the mean ± SD. Bars without the same letter differed significantly (*p* < 0.05). prot: protein.

The results presented in Figure 3 showed that the VLDLy in the serum of the VE is statistically similar to that of the Q group (*p* > 0.05), whereas Q and Q + VE are also statistically similar (*p* > 0.05). However, no difference existed between the control and vitamin E groups, as well as between the quercetin and combination (Q + VE) groups (*p* > 0.05). In addition, the VLDLy in the liver of the combination (Q + VE) group was higher as compared to the control group (*p* < 0.05). There was no difference observed between the control, quercetin and vitamin E groups, as well as between the quercetin, vitamin E, and the combination (Q + VE) groups (*p* > 0.05). The VLDLy level in the ovary of the combination (Q + VE) group was higher as compared to all the other groups (*p* < 0.05); however, no difference existed between the control, quercetin and vitamin E groups (*p* > 0.05, Figure 3A–C).

Furthermore, the VTG in the serum of the treatment groups were higher compared to the control group (*p* < 0.05). However, the combination (Q + VE) group was higher than the quercetin and the vitamin E groups (*p* < 0.05). No difference was observed between the quercetin and vitamin E groups. The liver VTG concentration in the Q + VE was higher as compared to the control group (*p* < 0.05). However, no difference was observed between the control, quercetin and vitamin E groups, as well as between the quercetin, vitamin E, and Q + VE groups (*p* > 0.05). There was no difference between the control, quercetin, and vitamin E groups, as well as between the quercetin, vitamin E, and combination (Q + VE) groups (*p* > 0.05). In addition, the ovary VTG level in the combination (Q + VE) group was higher compared to the other treatment groups (*p* < 0.05), however, there was no difference between the control, quercetin, and vitamin E groups (*p* > 0.05, Figure 3D–F).

### 3.4. Effects of Q, VE, and Q + VE on the Antioxidant Capacity and MDA Levels of the Liver, Blood, and Ovary of Aging Breeder Hens

#### 3.4.1. Antioxidant Capacity of the Liver, Blood, and Ovary of Aging Breeder Hens

**Figure 4:**
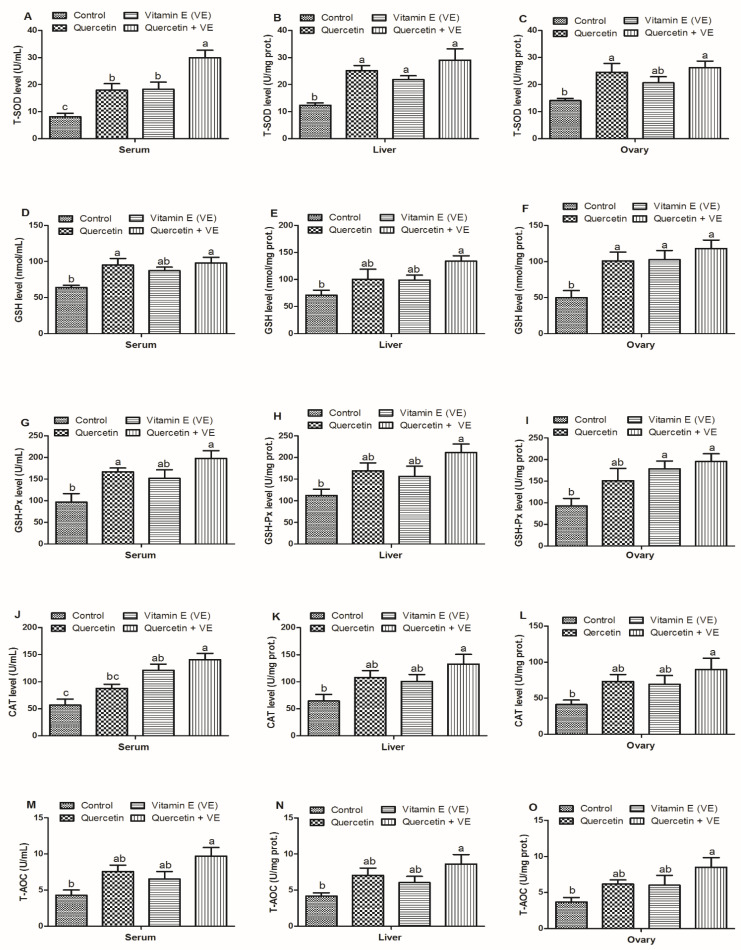
Effects of quercetin, vitamin E, and quercetin + vitamin E on the antioxidant capacity of the liver, blood and ovary of aging breeder hens. Levels of total antioxidant capacity (T-AOC) (**A**–**C**), concentrations of glutathione (GSH) (**D**–**F**), glutathione peroxidase (GSH-Px) (**G**–**I**)**,** catalase (CAT) (**J**–**L**), and total superoxide dismutase (T-SOD) (**M**–**O**). The values are presented as the mean ± SD. Bars without the same letter differed significantly (*p* < 0.05). prot: protein.

In Figure 4, the serum T-SOD levels were elevated in the treatment groups (*p* < 0.05); however, the combined (Q + VE) group was significantly higher than the quercetin and the vitamin E groups (*p* < 0.05). No difference was observed between the quercetin and the vitamin E groups (*p* > 0.05). In addition, the T-SOD levels in the liver tissues of the treatment groups were higher as compared to the control group (*p* < 0.05). The T-SOD levels in the ovary of the treatment groups were increased in the treatment groups as compared to the control group; however, the quercetin and the combination (Q + VE) groups showed significant difference with the control group (*p* < 0.05, Figure 4A–C). Moreover, the levels of GSH in the serum were dramatically increased in the combination group (Q + VE) as compared to the control group (*p* < 0.05), but are similar to the quercetin and vitamin E groups, whereas the VE and control groups are also similar (*p* > 0.05). A similar trend was observed in the levels of GSH in the liver (*p* < 0.05) but the quercetin and vitamin E groups were similar to the control (*p* > 0.05). Moreover, ovary GSH concentration is statistically similar (*p* > 0.05) in the Q, VE, and Q + VE groups as compared to the control group (*p* < 0.05, Figure 4D–F). 

Furthermore, the GSH-px levels in the serum were elevated in the treatment groups as compared to the control group; however, the quercetin and the combination (Q + VE) groups showed significant differences with the control group (*p* < 0.05). In addition, the GSH-px levels in the liver tissues were elevated in the treatment groups as compared to the control group; however, the combination (Q + VE) group showed significant differences with the control group (*p* < 0.05). Moreover, there were no difference existed between the control group compared with both the quercetin and vitamin E groups, as well as among all of the treatment groups (*p* > 0.05)..In the ovary, the GSH-px levels were increased in the treatment groups as compared to the control group; however, the vitamin E and combination (Q + VE) groups showed significant differences with the control group (*p* < 0.05). No difference was observed between control and quercetin groups (*p* > 0.05, Figure 4G–I). CAT activity in the serum was higher in the treatment groups, but the vitamin E and combination (Q + VE) groups were different compared to the control group (*p* < 0.05). There was no difference between the control and quercetin groups, as well as between the vitamin E and combination (Q + VE) groups (*p* > 0.05). In the liver and ovary, the CAT activity was elevated in the treatment groups as compared to the control group, but the combination (Q + VE) group showed a clear difference compared to the control group (*p* < 0.05). Furthermore, no difference existed between the control group compared with both the quercetin and vitamin E groups, as well as among all the treatment groups (*p* > 0.05, Figure 4J–L). Our results further showed that the T-AOC levels in the serum, liver and ovary were higher in the treatment groups as compared to the control group; however, only the combination (Q + VE) group was different compared to the control group (*p* < 0.05). We observed no difference between the control, quercetin and the vitamin E groups as well as between the quercetin, vitamin E and combination (Q + VE) groups (*p* > 0.05, Figure 4M–O). 

#### 3.4.2. MDA Levels in the Liver, Blood, and Ovary of Aging Breeder Hens

**Figure 5:**
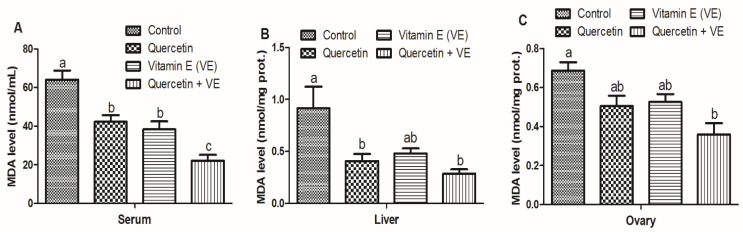
Effects of quercetin, vitamin E, and quercetin + vitamin E on the serum, liver, and ovary methane dicarboxylic aldehyde (MDA) level of aging breeder hens. Levels of methane dicarboxylic aldehyde (MDA) (**A**–**C**). The values are presented as the mean ± SD. Bars without the same letter differed significantly (*p* < 0.05). prot: protein.

The results shown in Figure 5 indicated that the MDA levels in the serum were reduced significantly in the treatment groups as compared to the control group (*p* < 0.05); however, they were significantly reduced in the combination (Q + VE) group as compared to the control, quercetin and vitamin E groups (*p* < 0.05). There was no difference between the quercetin and the vitamin E groups (*p* > 0.05, Figure 5A). For the liver tissues, the levels of MDA were also reduced as compared to the control, but the quercetin and the combination (Q + VE) groups were different compared to the control group (*p* < 0.05). There was no difference between the control group and the vitamin E group as well as between all the treatment groups (*p* > 0.05, Figure 5B). Lastly, the MDA levels in the ovary were reduced in the treatment groups as compared to the control group; however, the combination (Q + VE) group was significantly reduced as compared to the control group (*p* < 0.05). No difference occurred between the control, quercetin and vitamin E groups as well as between the quercetin, vitamin E and combination (Q + VE) groups (*p* > 0.05, Figure 5C). These results indicated that the liver, serum, and ovarian antioxidant capacity could be improved by the synergistic effects of combining quercetin + vitamin E supplementation in vivo in aging breeder hens. 

### 3.5. Effects of Quercetin (Q), Vitamin E (VE), and Their Combination (Q + VE) on Serum Hormone Levels of Aging Breeder Hens

**Figure 6:**
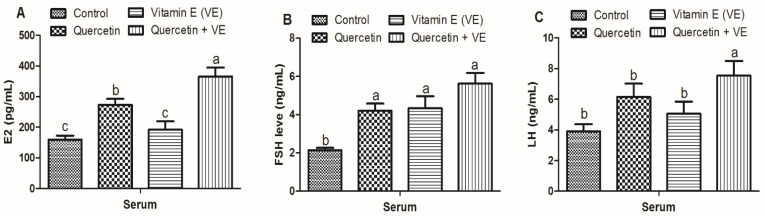
Effects of quercetin (Q), vitamin E (VE), and their combination (Q + VE) on serum hormone contents of aging breeder hens. Levels of E2 (**A**), FSH (**B**), and LH (**C**). The values are presented as the mean ± SD. Bars without the same letter differed significantly (*p* < 0.05).

The results presented in Figure 6 indicated that the levels of E2 in the serum of the combination (Q + VE) group were significantly higher as compared to the control, quercetin, and vitamin E groups (*p* < 0.05). However, the quercetin group was significantly increased as compared to the control and vitamin groups (*p* < 0.05, Figure 6A). In addition, the levels of FSH in the serum were significantly elevated in the treatment groups as compared to the control group (*p* < 0.05, Figure 6B). The LH level in the serum of the combination (Q + VE) group was elevated as compared to the control, quercetin, and vitamin E groups (*p* < 0.05). However, there was no significant difference between the control, quercetin and vitamin E groups (*p* > 0.05, Figure 6C). 

### 3.6. Effects of Q, VE, and Q + VE on the Levels of mRNA Expression of Hormone Receptors in the Ovary of Aging Breeder Hens

**Figure 7:**
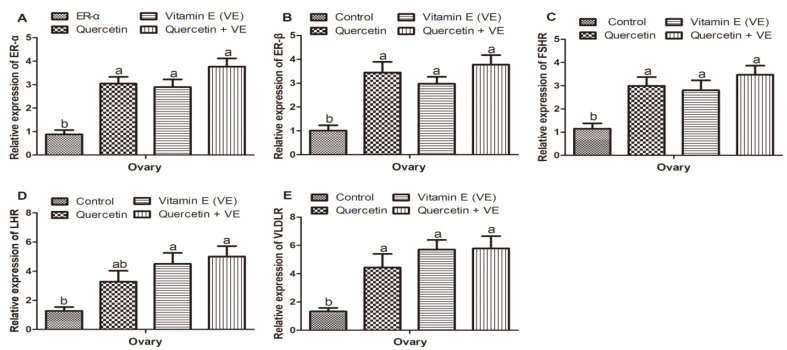
Effects of quercetin, vitamin E, and quercetin + vitamin E on the levels of mRNA expression of hormone receptors in the ovary of aging breeder hens. Levels of expression of *ER-α* (**A**), *ER-β* (**B**), *FSHR* (**C**), *LHR* (**D**), and *VLDLR* (**E**). The values are presented as the mean ± SD. Bars without the same letter differed significantly (*p* < 0.05).

Figure 7 summarizes the results of the mRNA expression of hormone receptors in the ovary. From the results, we observed that the mRNA expression of *ER-α*, and *ER-β* in the treatment groups was higher as compared to the control group (*p* < 0.05); however, no difference existed between the treatment groups (*p* > 0.05, Figure 7A,B). Similar trends were observed in the mRNA expression of *FSHR* and *VLDLR* in the ovaries of all the groups. The mRNA expression of *LHR* was increased in the treatment groups as compared to the control group, whereas the vitamin E and the combination (Q + VE) groups were significantly increased compared to the control group (*p* < 0.05). However, the expression of *LHR* was similar between the control and the quercetin groups, as well as between the quercetin, vitamin E, and combination (Q + VE) groups (*p* > 0.05, Figure 7C–E).

### 3.7. Effects of Q, VE, and Q + VE on the mRNA Expression of Liver ER-α and ER-β of the Aging Breeder Hens

**Figure 8:**
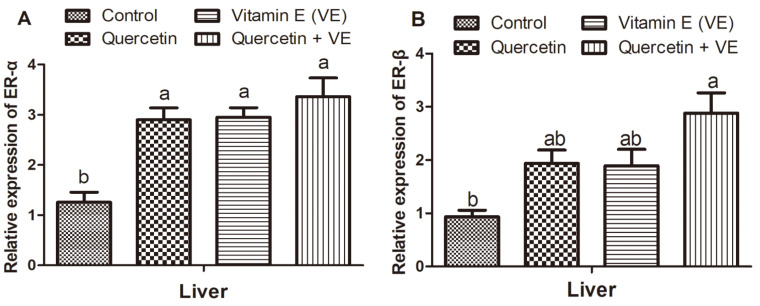
Effects of quercetin, vitamin E, and quercetin + vitamin E on the mRNA expression of liver *ER-α* and *ER-β* of the aging breeder hens. Levels of expression of *ER-α* (**A**) and *ER-β* (**B**). The values are presented as the mean ± SD. Bars without the same letters differed significantly (*p* < 0.05).

The gene expression of *ER-α* in the liver was high in the treatment groups as compared to the control group (*p* < 0.05), as shown in Figure 8A. There was no difference between the treatment groups (*p* > 0.05). For the expression of *ER-β* mRNA in the liver, the treatment groups had more elevated levels as compared to the control group, but levels in the combination (Q + VE) group were also increased significantly as compared to the control group (*p* < 0.05). No difference was observed between the control group compared with the quercetin and vitamin E groups, as well as among the quercetin, vitamin E and combination (Q + VE) groups (p > 0.05, Figure 8B).

### 3.8. Effects of Q, VE, and Q + VE on the Expression of mRNAs Related to Yolk Precursor Synthesis in the Liver of Aging Breeder Hens

**Figure 9:**
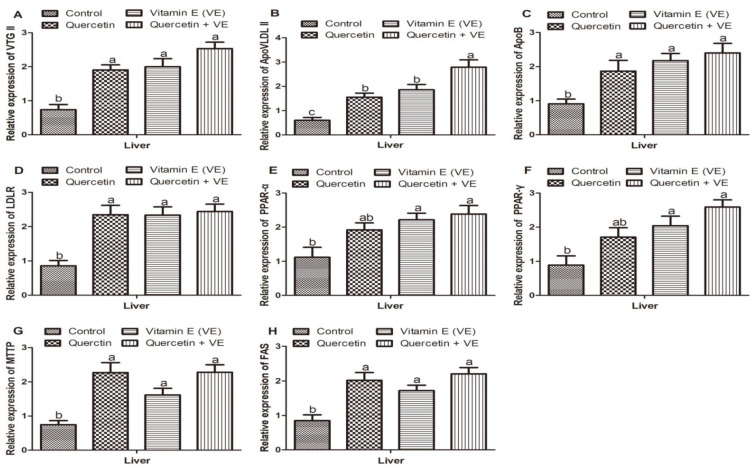
Effects of quercetin, vitamin E, and quercetin + vitamin E on the expression of mRNAs related to yolk precursor synthesis in the liver of aging breeder hens. Levels of expression of *VTG II* (**A**), *ApoVLDL II* (**B**), *ApoB* (**C**), *LDLR* (**D**), *PPAR-α* (**E**), *PPAR-γ* (**F**), *MTTP* (**G**), and *FAS* (**H**). Bars without the same letter differed significantly (*p* < 0.05).

Figure 9 shows the results of the transcription levels of genes related to yolk precursor synthesis in the aging breeder hens. The expression of *VTGII, ApoVLDLII*, *ApoB*, *LDLR*, *MTTP*, and *FAS* mRNAs in hen livers increased markedly in the quercetin, vitamin E, and the combination (Q + VE) groups without any difference between the supplementary groups, whereas the highest expressions were recorded in the combination group compared with the control group (*p* < 0.05, Figure 9A–D,G,H). The mRNA levels of peroxisome proliferator-activated α (*PPAR-α*) and peroxisome proliferator-activated γ (*PPAR-γ*) were similar in the control and the quercetin groups (*p* > 0.05), but, in comparison to the control, the vitamin E and combination groups differed significantly (*p* < 0.05), with the highest levels recorded in the combination group; however, the quercetin group was similar compared to the vitamin E and the combination groups (*p* > 0.05, Figure 9E,F). These findings demonstrated that during the aging process in breeder hens, the transcription levels of the critical genes related to yolk precursor synthesis were improved by the dietary supplements, with the highest expressions recorded in the combination group (Q + VE).

### 3.9. Effects of Q, VE, and Q + VE on the Expression of Apoptotic Genes in the Liver and Ovary of the Aging Breeder Hens

#### Relative Expression of Apoptosis-Related Genes in the Ovary and Liver

**Figure 10:**
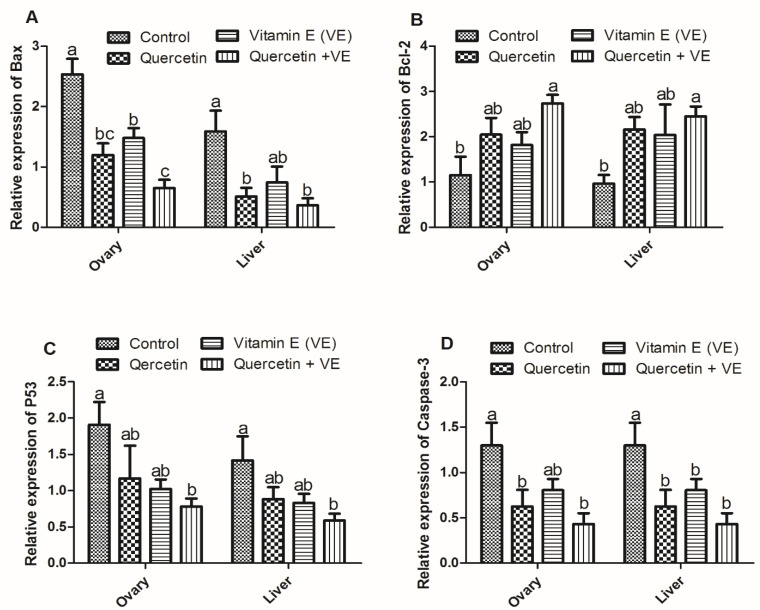
Effects of quercetin, vitamin E, and quercetin + vitamin E on the relative expression of apoptosis-related genes in the ovary and liver tissues. Levels of expression of *Bax* (**A**), *Bcl-2* (**B**), *p53* (**C**), and *Caspase-3* (**D**). Bars without the same letter differed significantly (*p* < 0.05).

From the result (Figure 10), it can be seen that the expression of *Bax* in the ovary tissues was reduced in the treatment groups as compared to the control group (*p* < 0.05). However, there was difference between the vitamin E and the combination (Q + VE) groups (*p* < 0.05). No difference existed between the quercetin and the vitamin E groups, as well as between the quercetin and the combination (Q + VE) groups (*p* > 0.05, Figure 10A). The expression of *Bax* in the liver tissues was reduced in the treatment groups as compared to the control group. However, the quercetin and the combination (Q + VE) groups were reduced significantly as compared to the control group (*p* < 0.05). The control and vitamin E groups were similar, as were the quercetin, vitamin E and the combination (Q + VE) groups (*p* > 0.05, Figure 10A). On the other hand, *Bcl-2* was increased in the treatment groups as compared to the control group, but the combination group increased significantly compared to the control (*p* < 0.05). There was no difference observed between the control, quercetin and the vitamin E groups, as well as between the quercetin, vitamin E and the combination (Q + VE) groups (*p* > 0.05, Figure 10B). On the other hand, *Bcl-2* was increased in the treatment groups as compared to the control group, but the combination group showed a significant increase compared to the control (*p* < 0.05). There was no difference observed between the control, quercetin and the vitamin E groups, as well as between the quercetin, vitamin E and the combination (Q + VE) groups (*p* > 0.05, Figure 10B). In addition, the mRNA expression level of *p53* in the ovary was decreased in the treatment groups as compared to the control group; however, significant differences were observed between the control and the combination (Q + VE) groups (*p* < 0.05). There was no difference observed between the control, quercetin, and the vitamin E groups, as well as between the quercetin, vitamin E, and the combination (Q + VE) groups (*p* > 0.05, Figure 10C). Furthermore, the mRNA expression level of *p53* in the liver was decreased in the treatment groups as compared to the control group; however, significant differences were observed between the control and the combination (Q + VE) groups (*p* < 0.05). There was no difference observed between the control, quercetin, and the vitamin E groups, as well as between the quercetin, vitamin E, and the combination (Q + VE) groups (*p* > 0.05, Figure 10C). *Caspase-3* expression levels in the ovary were reduced in the treatment groups as compared to the control group; however, significant differences were observed between the control and the combination (Q + VE) groups (*p* < 0.05). However, the control and vitamin E groups were similar, as well as the quercetin, vitamin E and the combination (Q + VE) groups (*p* > 0.05, Figure 10D). The expression of *Caspase-3* in the liver was reduced in the treatment groups as compared to the control group (*p* < 0.05), but quercetin, vitamin E, and the combination (Q + VE) groups were similar (*p* > 0.05, Figure 10D). 

### 3.10. Effects of Q, VE, and Q + VE on Serum AST and ALT Levels, and the mRNA Expression of Inflammation and Anti-Inflammation Related Cytokines in the Liver of Aging Breeder Hens

**Figure 11:**
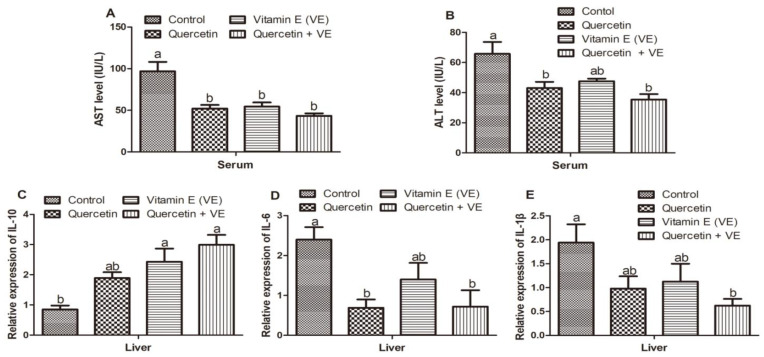
Effects of quercetin, vitamin E, and quercetin + vitamin E on serum AST and ALT levels, and the mRNA expression of inflammation and anti-inflammation related cytokines in the liver of aging breeder hens. Levels of AST (**A**), ALT (**B**), *IL-10* (**C**), *IL-6* (**D**), and *IL-1β* (**E**). The values are presented as the mean ± SD. Bars without the same letter differed significantly (*p* < 0.05).

From the results shown in Figure 11, the AST levels in the serum were reduced in all the dietary supplementary groups as compared to the control (*p* < 0.05). However, no difference existed between the quercetin, vitamin E, and combination groups (*p* > 0.05, Figure 11A). In addition, ALT levels in the control group were elevated as compared to the quercetin and combination (Q + VE) treatment groups (*p* < 0.05), but were not significantly different to the vitamin E group (*p* > 0.05, Figure 11B).

The mRNA expression of *IL-10* in the liver was increased in the dietary supplementary groups but decreased in the control group; however, levels in the vitamin E and combination groups were significantly increased compared to the control group (*p* < 0.05). No difference existed between the control and the quercetin groups, as well as between all the supplementary groups (*p* > 0.05, Figure 11C). Moreover, the mRNA expression of *IL-6* in the liver tissues was reduced in the treatment groups as compared to the control group; however, the quercetin and the combination groups were significantly reduced as compared to the control group (*p* < 0.05). No difference existed between the control and the vitamin E groups as well as among all the treatment groups (*p* > 0.05, Figure 11D). 

Finally, the mRNA expression of *IL-1β* in the liver tissues was reduced in the treatment groups as compared to the control group, but the combination group was significantly reduced as compared to the control group (*p* < 0.05). No difference existed between the control group compared with both the quercetin and vitamin E groups, as well as among all of the treatment groups (*p* > 0.05, Figure 11E). 

## 4. Discussion

In the present study, we investigated the effects of supplementation with quercetin (Q), vitamin E (VE), and the combination of both (Q + VE) on the reproductive organ characteristics and yolk precursor formation in the liver by comparing TG, T-CHO, VLDLy, and VTG levels in the liver–blood–ovary signal axis. We also determined the dietary effects on the antioxidant capacity of the liver, serum, and ovary; liver morphological and histological changes; serum reproductive hormone levels; the mRNA expression of the reproductive hormone receptors; as well as the expression of genes related to yolk precursor synthesis. Furthermore, serum aspartate aminotransferase (AST) and alanine aminotransferase (ALT) (used to assess hepatocellular injury), and mRNAs related to apoptosis and inflammation, were determined in the aging breeder hens. 

Reports vividly indicate that supplementing dietary antioxidants attenuates oxidative stress and inflammation in organs including the liver and ovary, thereby serving as an anti-aging compound [18,40,41]. Quercetin and vitamin E exhibit several biological properties including anti-aging, anti-oxidative, anti-inflammatory, phyto-estrogenic, and anti-apoptotic characteristics in vivo [27,42]. Dietary vitamin E has been found to improve the reproduction and antioxidant capability of breeder chickens [31,32]. Reports have indicated that vitamin E protects the liver from lipid peroxidation and prevents cell membrane damage. Antioxidants play an important role in avian reproduction [30]. In the present study, quercetin and vitamin E, and to a larger extent the combination of both (Q + VE), improved the characteristics of the reproductive organs such as the follicle numbers and weights, and the uterus, ovary and liver weights. However, these results are inconsistent with previous reports that used dietary quercetin or vitamin E separately and, hence, reported no significant differences between organ characteristics such as those of the ovary, uterus, follicles, and liver [27,30]. However, the difference between the present study and previous reports may be due to the differences in chicken age and the combined supplements (Q + VE) used in this study. This indicates that the estrogen-like activity of the combined quercetin and vitamin E resulted in improved organ growth and development. The increase in follicular number by the combination of quercetin and vitamin E indicated that they could synergistically reduce the proportion of follicular atresia, thereby improving fertilization and laying performance in aging breeder hens. The number of hierarchical follicles indicates the reproductive status of the aging breeder hens [3]. 

Growing evidence suggests that quercetin and vitamin E play major roles in the prevention of tissue pathologies such as inflammation, oxidative stress, and autoimmune disorders [42,43]. In this study, the liver morphology and histology were improved massively by the combined efforts of quercetin and vitamin E; thus, there were no hepatic steatosis or fatty liver conditions observed in the Q + VE groups as assessed by HE and oil red staining. This shows that combining quercetin and vitamin E exerted a synergetic protective effect on liver morphological damage during the aging process. This was consistent with the changes observed in the serum AST and ALT levels, as well as the mRNA expression of the inflammatory cytokines (*IL-6* and *IL-1β*) in the liver tissue, which were lower in the combination group (Q + VE) compared to the control; however, the anti-inflammatory cytokine (*IL-10*) was elevated in the combination group, indicating that combining quercetin and vitamin E could attenuate inflammation and oxidative stress in the aging hens, hence preventing liver damage, as confirmed by the results of HE and oil red staining.

These results are in accordance with those reported by Liu et al. (2018), insofar as antioxidants such as lycopene and grape seed proanthocyanidin extract could exert protective effects on liver cells, thereby preventing hepatic steatosis in chickens. The results also conformed to the findings of [44].

Reports suggest that the processes of lipogenesis and vitellogenesis may decrease with increasing age in breeder chickens, as indicated by changes in the liver, blood, and ovary biochemical and antioxidant levels, serum reproductive hormone levels, and expression of mRNAs related to the synthesis of yolk precursors [3]. In chickens, the lipid and protein profiles are represented as triglycerides (TG), and VLDL and VTG, respectively, which are mainly synthesized in the hepatocytes, and stored in the growing oocytes that eventually promote follicle development [3]. The protein portions of the yolk precursors are synthesized under the influence of estrogen and progesterone. In this study, the increased number of follicles, and the levels of TG in the liver, serum, and ovary of the hens fed the combination of quercetin and vitamin E, as compared to other groups, showed that the supplementation of quercetin + vitamin E could synergistically promote yolk precursor synthesis in the aging hens, as evidenced by the levels of expression of TG, VTG, and VLDL obtained in this study. These results are consistent with previous reports that indicated that dietary supplementation with vitamin E improves egg production by facilitating the release of vitellogenin (VTG) from the liver and increasing its concentration in the blood stream [35]. Phyto-estrogens can bind to estrogen receptors and, hence, can activate estrogen-related mRNAs [27]. Quercetin, a major phyto-estrogenic compound, has both estrogenic and anti-estrogenic actions, thereby promoting the secretion and concentration of reproductive hormones and hormone receptors, especially estrogen [45]. The molecular structures of quercetin and estradiol are similar; hence, they can easily function as estrogen agonists and antagonists [46]. As evidenced in this study, the elevated levels of serum-reproductive hormones such as E2, FSH, LH, and their respective receptors (*ERα*, *ERβ*, *FSHR*, and *LHR*) in the liver and ovary of the aging hens fed quercetin + vitamin E, as compared to the control group, indicated that during the aging process in breeder hens, quercetin + vitamin could synergistically promote hormone synthesis that could directly influence follicle development. Hence, it is clear in this study that in the combination group (Q + VE), the increased levels of estradiol and its receptors influenced the elevated mRNA expressions of *ApoB*, *VTG II*, *ApoVLDL II*, and *LDLR*, and the changes in TG levels were consistent with the expression of the mRNAs related to lipolysis (*PPAR-γ*, *PPARα*), lipogenesis (*FAS*), and lipid transport (*MTTP*), which significantly contributed to yolk precursor synthesis. These results are consistent with those reported by [27] and [47], indicating that quercetin acts as a phyto-estrogen that influences the transcription of genes and receptors related to yolk precursor synthesis in birds. Another report explained that estrogen-like activity of phyto-estrogen promotes the secretion of prolactin (PRL), insulin-like growth factor-1 (IGF-1), and GH [48]. The results obtained in this study also conformed with the previous findings that in aging hens, the mRNA levels of *ApoVLDLII* and *ApoB* were elevated following the release of estrogen post laying [49,50]. Hence, during the aging process, the combined effects of quercetin and vitamin E exerted strong estrogen-like activity that promotes the transcription of yolk precursors, which also influences organ characteristics, thereby promoting follicle development in the aging breeder hens. 

Dynamics in organ systems result in the accumulation of reactive oxygen species (ROS); however, the presence of an active enzymatic antioxidant system in cells attenuates excessive accumulation of ROS, thereby preventing oxidative stress [51]. However, in most aging animals, ROS accumulation overrides the antioxidant capacity of the cells due to the reduction in cell numbers and concentration of antioxidant enzymes, resulting in oxidative stress [19], which can cause severe changes in the cytoplasm and nuclear regions of oocytes and other organs, such as the liver, which subsequently may affect yolk precursor production and the fertilization capacity of oocytes [18,51]. Quercetin has been shown to have a protective effect on pre-ovulatory follicles by promoting antioxidant capacity and reducing apoptosis in granulosa cells, thereby promoting follicle development in rabbits under heat stress [52]. In the present study, we observed that the MDA levels were significantly reduced in the combination group (Q + VE) as compared to the other groups, and antioxidant enzymes such as CAT, T-SOD, GSH, GSH-Px, and T-AOC were dramatically increased by the synergistic effects of quercetin + vitamin E in the liver, serum, and ovary of the aging hens. These results are consistent with those reported by Dong et al. (2020), who stated that quercetin significantly upregulated the transcription of nuclear factor erythroid 2–related factor 2 (Nrf2) and its downstream genes such as catalase, superoxide dismutase, glutathione peroxidase 2, heme-oxygenase-1 (HO-1), and thioredoxin, thereby ameliorating oxidative stress in chickens fed with oxidized oil. Liu et al. (2019) also reported that vitamin E supplementation alleviates the oxidative and immune stress induced by *Salmonella Enteritidis* challenge by reducing MDA levels and serum inflammatory cytokines (*IL-6* and *IL-1β*). Moreover, in this study, the expression levels of apoptotic genes such as *Bax*, *caspase-3*, and *p53* were reduced significantly, whereas the expression of *Bcl-2* was increased in the liver and ovary tissues of aging hens fed with quercetin + vitamin E. These results are consistent with previous reports that indicated that the enzymatic antioxidant system could halt oxidative stress [50,53], and Yang et al. (2018) reported that quercetin supplementation reduces apoptosis in laying hens. Other reports indicated that quercetin exhibited protective effects on cumulus cells by maintaining their antioxidant capacity against cadmium-induced toxicity, and also prevented apoptosis in the cells by reducing the expression of *Bax* and *caspase-3* [54]. Furthermore, reports have indicated that high expression of *Bcl-2* correlates with estrogen secretion and the expression of estrogen receptors [27], which was confirmed in this study as the combination of quercetin and vitamin E exerted estrogen-like effects by increasing the secretion of estrogen and the expression of its receptors, thereby inhibiting apoptosis. These results indicated that the estrogen-like effect of quercetin + vitamin E inhibited atresia in the liver and ovary of the aging breeder hens, as shown in the liver HE and oil red staining results. In this study, we showed that supplementation with quercetin + vitamin E effectively promoted follicle development and alleviated oxidative stress, inflammation, and apoptosis in aging breeder hens.

## 5. Conclusions

**Figure 12:**
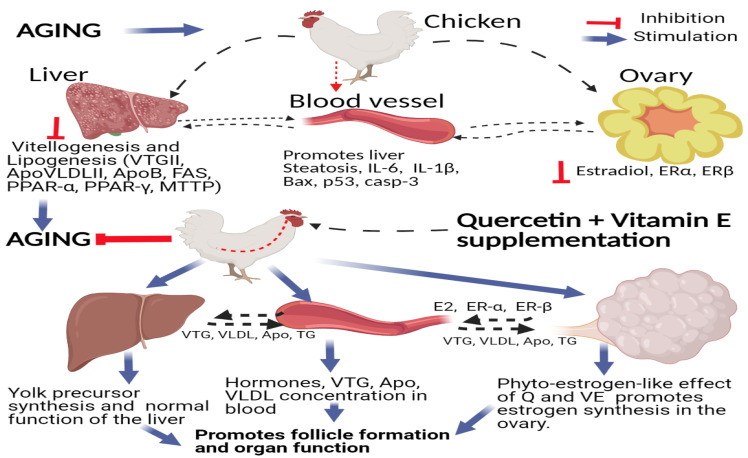
Schematic diagram summarizing the mechanisms underlying the hepatoprotective and oviprotective effects of the combination of quercetin and vitamin E to promote follicle formation and attenuate oxidative stress, inflammation, and apoptosis in aging breeder hens. Supplementation of quercetin + vitamin E promotes the synthesis and expression of reproductive hormones and their receptors, and up-regulates the transcriptions related to vitellogenesis and lipogenesis via the liver–blood–ovary signal axis in aging breeder hens.

In conclusion, in this study, we observed that dietary supplementation with quercetin and vitamin E increased the reproductive performance of aging breeder hens, via the liver–blood–ovary signal axis, by increasing the secretion of hormones and their receptors, promoting the development of reproductive organs, inhibiting ovarian and liver apoptosis, and promoting transcription of mRNAs related to yolk precursor synthesis, thereby promoting follicle development and fecundity. These results provide an important reference for the combination of quercetin and vitamin E as a functional feed additive for promoting the functions of the liver–blood–ovary axis, and as a potential chemopreventive and chemotherapeutic agent for improving liver and ovary functions in chickens by acting as hepatoprotective and oviprotective agents. Therefore, the combination of quercetin and vitamin E synergistically promotes and regulates the transportation and exchange of synthetic substances (including hormones, yolk precursors, and other biochemical substances) among the liver–blood–ovary alliances to ensure synchronous development and functional coordination between the liver and ovary in aging breeder hens (Figure 12). 

## Figures and Tables

**Table 1:** Ingredient composition and calculated nutrient content of basal diet (% Dry matter).

Ingredient	Content (%)	Nutrient	%
Corn	56.4	Metabolic Energy (ME)	10.98 MJ/kg
Soybean meal	26.2	Crude protein	17.04
Wheat bran	2.4	Crude fat	3.47
Corn germ meal	3.5	Crude fibre	2.68
Lard	0.8	Calcium	3.4
Limestone (fine)	2.3	Total phosphorus	0.63
Limestone (coarse)	6	Available phosphorus	0.37
Dicalcium phosphate	1.34	Lysine	0.86
Sodium chloride	0.24	Methionine	0.39
Choline chloride	0.12		
Vitamin premix ^1^ + mineral premix ^2^	0.7		
Total	100		

Vitamin premix ^1^ supplied (per kg of diet): Vitamin A, 6000 IU; Vitamin D3, 1500 IU; Vitamin K3, 4.2 mg; Vitamin B1, 3 mg; Vitamin B2, 10.2 mg; Folic acid, 0.9 mg; Calcium pantothenate, 15 mg; Niacin 45 mg; Vitamin B6, 5.4 mg; Vitamin B12, 24 μg; Biotin 150 μg. Mineral premix ^2^ provided (per kg of diet): Cu (CuSO_4_·5H_2_O), 6.8 mg; Fe (FeSO_4_·7H_2_O), 66 mg; Zn (ZnSO_4_·7H_2_O), 83 mg; Mn (MnSO_4_·H_2_O), 80 mg; I (KI), 1 mg; Se (Na_2_SeO_3_), 0.3 mg.

**Table 2:** Primers used for quantitative real-time polymerase chain reaction (qRT-PCR).

Gene	Sequence (5′-3′)	Product Length (bp)	Annealing Temperature (℃)	Accession Number
*FSHR*	F: ACCACACGTGCCTCTGTGAA R: GCTCCCTTCGGAATGACTCT	136	59.17	NM_205079.1
*LHR*	F: CGGATACACAACGATGCCCT R: TTTATCCAGAGGCGGCAGTC	159	59.82	NM_204936.1
*VLDLR/LR8*	F: AACGAGGCAGTCTATGGTGC R: TGTTGAATCCTCCACATCTCAG	271	57.79	NM_205229.1
*VTGII*	F: AACTACTCGATGCCCGCAAA R: ACCAGCAGTTTCACCTGTCC	179	58	NM_001031276.1
*ApoVLDL-II*	F: CCTTAGCACCACTGTCCCTG R: AGCTCTAGGGGACACCTTGT	130	58	NM_205483.2
*ApoB*	F: ACACTTCGGGCTATTGGA R: TGCCTGTATGGCTGCTTT	129	60	NM_001044633.1
*LDLR*	F: GGGAACCTCTATTGGGCCG R: CAACATGGGATCCAACGCGA	270	59.85	NM_204452.1
*ER-α*	F: TGTGCTGTGTGCAACGACTA R: CAGGCCTGGCAACTCTTTCT	167	57	NM_205183.2
*ER-β*	F: GGCTGCAACCCGTGTAAAAG R: GCCCAGCCAATCATGTGAAC	189	58	NM_204794.2
*PPAR-α*	F: AGGCCAAGTTGAAAGCAGAA R: TTTCCCTGCAAGGATGACTC	155	60	NM_001001464.1
*PPAR-γ*	F: TGACAGGAAAGACGACAGACA R: CTCCACAGAGCGAAACTGAC	164	59	NM_001001460.1
*MTTP*	F: GTTCTGAAGGACATGCGTGC R: GATGTCTAGGCCGTACGTGG	120	58	NM_001109784.2
*FAS*	F: GCTAAGATGGCATTGCACGG R: TCCATTCAGTTCCAGACGGC	135	58	NM_205155.1
*Bcl- 2*	F: ATCGTCGCCTTCTTCGAGTT R: ATCCCATCCTCCGTTGTCCT	150	59	Z11961.1
*Bax*	F: GTGATGGCATGGGACATAGCTC R: TGGCGTAGACCTTGCGGATAA	90	58	XM_422067.4
*Caspase-3*	F: ACTCTGGAATTCTGCCTGATGACA R: CATCTGCATCCGTGCCTGA	129	59	NM_204725.1
*p53*	F:GAGATGCTGAAGGAGATCAATGAG R: GTGGTCAGTCCGAGCCTTTT	145	58	X13057.1
*IL-10*	F: GGAGAGAGCGGAGGTTTCG R: TCCCGTTCTCATCCATCTGC	118	59.86	XM_025143715.1
*IL-6*	F: CTGCAGGACGAGATGTGCAA R: AGGTCTGAAAGGCGAACAGG	175	60.67	NM_204628.1
*IL-1β*	F: TGCCTGCAGAAGAAGCCTCG R: GACGGGCTCAAAAACCTCCT	204	60.25	NM_204524.1
*GAPDH*	F: TCCTCCACCTTTGATGCG R: GTGCCTGGCTCACTCCTT	144	60	NM_204305.1

**Table 3:** Effects of dietary quercetin (Q), vitamin E (VE), and their combination (Q + VE) on the reproductive organ characteristics of aging breeder hens.

Parameters	Control	Quercetin	Vitamin E	Q + VE
Live Body weight (g)	2602.81 ± 11.96	2855.94 ± 310.09	2637.50 ± 223.17	2675.00 ± 214.41
Oviduct index (%)	2.15 ± 0.62 ^c^	2.57 ± 0.45 ^ac^	2.05 ± 0.56 ^ac^	2.75 ± 0.52 ^a^
Oviduct length (cm)	53.43 ± 9.63 ^b^	63.71 ± 10.10^a^	57.89 ± 7.54 ^ab^	64.60 ± 9.06 ^a^
Liver index (%)	2.10 ± 0.54	2.32 ± 0.43	0.32 ± 0.57	2.55 ± 0.69
Ovary index (%)	1.58 ± 0.41 ^b^	2.35 ± 0.52 ^a^	2.24 ± 0.52 ^a^	2.32 ± 0.53 ^a^
Follicle No. (F1-F3, >8 mm in diameter)	5.25 ± 1.48 ^b^	7.31 ± 1.99 ^a^	7.13 ± 1.02 ^a^	7.38 ± 1.09 ^a^
Follicle F1 index (%)	0.51 ± 0.15 ^b^	0.65 ± 0.08 ^ab^	0.67 ± 0.17 ^a^	0.71 ± 0.18 ^a^
Follicle F2 index (%)	0.31 ± 0.14 ^b^	0.53 ± 0.09 ^a^	0.50 ± 0.08 ^a^	0.57 ± 0.21 ^a^
Follicle F3 index (%)	0.20 ± 0.09 ^b^	0.32 ± 0.09 ^a^	0.30 ± 0.07 ^a^	0.32 ± 0.10 ^a^
Follicle diameter (mm) F1	28.74 ± 5.56 ^c^	34.83 ± 5.09 ^b^	33.54 ± 0.89 ^b^	39.57 ± 4.12 ^a^
Follicle diameter (mm) F2	22.90 ± 4.93 ^c^	31.19 ± 4.84 ^b^	30.07 ± 4.12 ^b^	35.06 ± 4.73 ^a^
Follicle diameter (mm) F3	20.23 ± 5.85 ^c^	26.48 ± 3.84 ^b^	25.55 ± 3.83 ^b^	31.47 ± 3.48 ^a^
Abdominal fat index (%)	4.60 ± 1.55	3.73 ± 1.94	3.90 ± 3.10	2.82 ± 1.92

^a–c^ Mean ± Standard Deviation values within the same row sharing a common superscript letter are not statistically different at *p* < 0.05 (*n* = 8 per group).

## Data Availability

The data presented in this study are available on request from the corresponding author.
